# Chronic Tinnitus and the Positive Effects of Sound Treatment via a Smartphone App: Mixed-Design Study

**DOI:** 10.2196/33543

**Published:** 2022-04-21

**Authors:** Justyna Jolanta Kutyba, W Wiktor Jędrzejczak, Elżbieta Gos, Danuta Raj-Koziak, Piotr Henryk Skarzynski

**Affiliations:** 1 World Hearing Center Institute of Physiology and Pathology of Hearing Warsaw Poland; 2 Institute of Sensory Organs Kajetany Poland; 3 Department of Heart Failure and Cardiac Rehabilitation, Second Faculty Medical University of Warsaw Warsaw Poland

**Keywords:** tinnitus, mobile app, smartphone, sound therapy, telemedicine, mobile phone

## Abstract

**Background:**

Tinnitus is a phantom auditory sensation in the absence of an external stimulus. It is accompanied by a broad range of negative emotional symptoms and a significantly lower quality of life. So far, there is no cure for tinnitus, although various treatment options have been tried. One of them is mobile technology employing dedicated apps based on sound therapy. The apps can be managed by the patient and tailored according to their needs.

**Objective:**

The study aims to assess the effect of a mobile app that generates background sounds on the severity of tinnitus.

**Methods:**

The study involved 68 adults who had chronic tinnitus. Participants were divided into a study group (44 patients) and a control group (24 patients). For 6 months those in the study group used a free mobile app that enriched the sound environment with a background sound. Participants were instructed to use the app for at least 30 minutes a day using their preferred sound. The participants in the control group did not use the app. Subjective changes in the day-to-day functioning of both groups were evaluated using the Tinnitus Handicap Inventory (THI) questionnaire, a visual analog scale, and a user survey.

**Results:**

After 3 months of using the app, the THI global score significantly decreased (*P*<.001) in the study group, decreasing again at 6 months (*P*<.001). The largest improvements were observed in the emotional and catastrophic reactions subscales. A clinically important change in the THI was reported by 39% of the study group (17/44). Almost 90% of the study participants (39/44) chose environmental sounds to listen to, the most popular being rain and ocean waves. In the control group, tinnitus severity did not change over 3 or 6 months.

**Conclusions:**

Although the participants still experienced limitations caused by tinnitus, the advantage of the app was that it led to lower negative emotions and thus reduced overall tinnitus severity. It is worth considering whether a mobile app might be incorporated into the management of tinnitus in a professional setting.

## Introduction

The digital revolution initiated in the 20th century has impacted many fields of life, including those related to health [1]. The use of technological solutions to improve treatments can be seen in every branch of medicine. Examples are solutions with social benefits, such as e-referrals and e-prescriptions [2], as well as those with individual benefits such as health monitoring wristbands [3] and virtual assistants [4]. Modern solutions are evident in cardiology [5], diabetology [6], and audiology [7], including patients with tinnitus.

Tinnitus is a sound that occurs without physical external stimulation and in most cases is heard only subjectively [8,9]. Tinnitus can be described as a squeaking, buzzing, humming, or clicking, either in the ears or in the head [8,10]. Epidemiology suggests the problem affects between 4% and 15% of the adult population and shows an increasing trend [10,11].

Currently, there is no established effective treatment. Experts dealing with tinnitus most often recommend sound therapy, which is the use of additional background sounds to change the patient’s reaction to tinnitus [10]. Sound therapy does not cure the condition, but may significantly lower its severity, reducing the level of distress or impact that tinnitus has on the individual [10]. The chronic nature of tinnitus often prompts patients to use various forms of self-help [10,12]. Studies have shown that self-help programs can be effective and provide benefits to patients in managing their tinnitus [13-15]. It has also been documented that one of the most commonly used forms of self-help is acoustic stimulation, that is, surrounding oneself with various types of sounds [13,16]. Sound enrichment causes the patient’s tinnitus to blend in with the environment and become less noticeable, thus improving overall comfort [17,18]. To enrich the auditory background, patients can use professional devices such as sound generators or broadband noise generators, as well as recorded CDs, the radio, and recently, mobile app [10,19].

In the mobile solutions market, a range of apps have been developed for people with tinnitus [20-22]: tutorials, apps containing information about tinnitus, apps to support psychotherapy, and others to gauge tinnitus characteristics or track tinnitus intensity throughout the day [23]. Studies show that the most commonly used apps generate sounds of nature, of everyday life, or relaxing music [23]. Patients use the apps because they are mostly free and easily available, and their variety makes it easy to select one that suits the individual.

The use of mobile apps in the treatment of tinnitus has been of interest to researchers for several years. There have been some papers describing the effects that mobile apps which generate sounds have on tinnitus. Henry et al [24] conducted a study on a group of 25 individuals who used a mobile sound-generating app for 8 weeks. The changes in tinnitus sensation as assessed by the Tinnitus Functional Index (TFI) questionnaire were generally small, although 8 of the 25 individuals achieved a reduction in total score of 13 points or more. This degree of reduction reflects a change that is likely to be clinically important to the patient.

Another study of app use was conducted in 2016 by Tyler et al [25] in a group of 16 individuals with a cochlear implant. The study assessed the level of acceptance of the sounds generated from the app. The participants reported that using an app was satisfactory and more comfortable than using tablets or computers. The participants indicated that the app provided them with a wide range of sounds and that the sounds provided to the implant were acceptable and pleasant.

In 2020 the current authors conducted a pilot study on 52 patients with tinnitus [26]. Participants listened to the mobile app daily for 6 months. Two questionnaires (TFI and Tinnitus Handicap Inventory [THI]) were used to evaluate the effect of the app on tinnitus severity. As gauged by both questionnaires, a significant reduction in tinnitus severity was found. Overall severity decreased after the first 3 months and again over the subsequent 3 months.

Although all these results are promising, these studies require further substantiation because only small groups of patients were studied, standardized tinnitus instruments were not always used, and there were no control groups. Thus, this study aimed to assess the effect of using a smartphone app that generates acoustic signals on the severity of tinnitus. The effect was evaluated by validated tinnitus-specific questionnaires after 3 and 6 months of use, and the results were compared with those obtained from people with tinnitus who did not use the app.

## Methods

### Participants and Setting

The participants were patients admitted to our tertiary referral center due to tinnitus. Only adult patients who had tinnitus for at least 6 months were recruited. The study’s purpose and procedures were explained to all potential participants. Afterward, those who were willing to participate and owned an Android or iOS smartphone were included in the study group; those who did not own an appropriate device or did not want to participate in the study group but wanted to have their tinnitus monitored were included in the control group.

A total of 147 participants were initially enrolled in the study, but the final analysis included results from 68 participants, made up of 44 in the study group and 24 in the control group. There were 79 participants who were excluded, made up of 50 patients initially recruited to the study group and 29 patients recruited to the control group. They were excluded due to the following reasons: (1) 52 (30 enrolled in the study group and 22 in the control group) did not send back the questionnaires; (2) 17 (14 enrolled in the study group and 3 in the control group) sent back questionnaires with incomplete responses and were unsuitable for further analysis; (3) 10 (6 enrolled in the study group and 4 in the control group) admitted they had used other forms of therapy while participating in the study.

The dropout rate was similar in both groups: 53% (50/94) in the study group and 55% (29/53) in the control group. In the study group the dropouts were of similar age (mean 52.7 [SD 13.6]) as those who completed the study (mean 51.9 [SD 11.6]; *t*_92_=0.30, *P*=.77). Tinnitus severity as measured with the THI global score was similar in the dropouts (mean 54.9 [SD 26.6]) and in the completers (mean 54.9 [SD 23.9]; *t*_92_=0.02; *P*=.99). Their results on all 3 THI subscales were also similar in the study group.

The same was true for the control group. The dropouts were of similar age (mean 51.6 [SD 13.0]) as those who completed the study (mean 51.9 [SD 14.1]; *t*_51_=0.09; *P*=.93). Tinnitus severity as measured with the THI global score was similar in the dropouts (mean 43.7 [SD 22.3]) and in the completers (mean 52.1 [SD 23.1]; *t*_51_=0.1.32; *P*=.19). Besides, their results on all 3 THI subscales were similar in the control group.

Patients in the study and control groups did not differ significantly in terms of sociodemographic data (Table 1).

**Table 1:** Characteristics of the participants.

Characteristics			Study group (n=44)		Control group (n=24)		Test result
**Age (years)**						*t*_66_=0.01; *P*=.99	
	Mean (SD)	51.9 (11.6)		51.9 (14.1)			
	Range	26-72		28-74			
**Sex, n (%)**						*χ*_1_^2^=0.04; *P*=.83	
	Female	25 (57)		13 (54)			
	Male	19 (43)		11 (46)			
**Educational status, n (%)^a^**						*χ*_1_^2^=0.14; *P*=.71	
	No higher education	19 (45)		12 (50)			
	Higher education	23 (55)		12 (50)			
**Place of residence, n (%)**						*χ*_1_^2^=0.68; *P*=.41	
	Rural	9 (20)		3 (13)			
	Urban	35 (80)		21 (87)			
**Tinnitus duration (years)**						*t*_66_=0.82; *P*=.42	
	Mean (SD)	6.2 (8.1)		4.7 (5.4)			
	Range	0.7-40		1-26			
**Tinnitus localization, n (%)^b^**						*χ*_1_^2^=2.57; *P*=.11	
	One ear	13 (30)		12 (50)			
	Both ears	30 (70)		12 (50)			
**Manifestation over time, n (%)^b^**						*χ*_1_^2^=0.04; *P*=.84	
	Constant	40 (93)		22 (92)			
	Intermittent	3 (7)		2 (8)			
**Hearing loss, n (%)**						*χ*_1_^2^=0.11; *P*=.74	
	Yes	22 (50)		13 (54)			
	No	22 (50)		11 (46)			

^a^n=42 for the study group because 2 participants did not state this.

^b^n=43 for the study group because 1 participant did not state this.

### ReSound Tinnitus Relief App

The ReSound Tinnitus Relief app was tested in our pilot study, and because of promising results, it was used again in this study [26,27]. The app was created in 2014 and is available on the Google Play Store and the App Store. Before starting the study, we tested the operation of the app on both platforms. As the operation of the app on both platforms was the same, in our study we did not control which operating system the participants used. This particular app was chosen because it is free, has a menu in Polish, and has already been described in the literature [24,25,27,28]. The main function of the app is to generate various acoustic signals. It is equipped with a library of 33 sounds divided into 3 categories: environment, music, and therapeutic sounds. In addition, it offers meditation support, a set of relaxation exercises, and a panel with information about tinnitus. Participants included in the study group were given the same recommendations for using the app as those in the pilot study [26]: duration of stimulation should be a minimum of 30 minutes per day and the sound should be emitted in free field and at a level slightly lower than the patient’s tinnitus. Patients decided for themselves which sounds they would listen to [26]. The patients in the control group did not use the app.

### Measures

To assess the patients, the Tinnitus Handicap Inventory, a visual analog scale (VAS), and a survey were used.

The THI is a standardized questionnaire to assess the impact of tinnitus on a patient’s daily functioning [29]. It is composed of 25 questions divided into 3 subscales: Functional, Emotional, and Catastrophic. For each question, the patient can answer *yes, sometimes,* or *no*. The maximum score is 100 points; the higher the score, the more severe the tinnitus. A reduction in total score of 20 or more points is considered a clinically significant change in tinnitus severity [30]. The Polish version of the questionnaire was used in this study; its psychometric values have been determined by Skarżyński et al [31,32].

VAS is a tool that allows the assessment of a patient’s tinnitus [33]. It presents a 10-cm-long line printed on a sheet of paper, with the ends of the line labeled “minimally” (at the left) and “maximally” (at the right). To calculate a VAS score, the length of the segment from the beginning of the line to the point indicated by the patient is measured and multiplied by 10, giving a number between 0 and 100. In this way, patients are asked to assess their level of tinnitus loudness and tinnitus annoyance. The VAS has been used in other studies of tinnitus [34-36].

A user survey was developed to assess how the participants used the app. The questions concerned the amount of time of using the app, the type of sounds listened to, subjective assessment of its effectiveness, and the use of other concurrent forms of therapy. The controls were given a modified version of the survey without questions relating to the app. The questions asked were: *How often do you use the application? How long do you use the application during the day? Have you made any other efforts at therapy while using the application?* Participants who answered “yes” to using other forms of therapy (n=10) were not included in the final analysis.

### Patient Assessment Procedure

All patients signed an informed consent form to participate in the study. Afterward, a medical examination and pure tone audiometry were conducted. The app was installed on the patient’s smartphone, information on how to use it was provided, and the patients filled in the THI and VAS questionnaires. Then, 3 and 6 months later, the participants received another set of questionnaires in the postal mail consisting of the THI, VAS, and the survey, and were again asked to fill them in and send them back.

### Statistical Analysis

The demographic and clinical characteristics of the study and control groups were examined using descriptive statistics and percentages. Differences across groups were assessed through an independent (unpaired) *t* test or a *χ*^2^ test. A mixed-design ANOVA with Bonferroni adjustment for multiple comparisons was used to evaluate tinnitus severity, loudness, and annoyance in the study and control groups over 6 months. A *P* value <.05 was considered statistically significant. The analysis was conducted using IBM SPSS Statistics (version 24).

### Ethics Approval

The study protocol was based on a previously conducted pilot study [26] and was approved by the Bioethics Committee of the Institute of Hearing Physiology and Pathology (KB. IFPS:15/2020).

## Results

### Tinnitus Severity as Measured With the THI

Descriptive statistics for the THI scores for initial and follow-up measurements in the study and control groups are shown in Tables 2 and 3.

A mixed-design ANOVA revealed that an interaction effect (group × time) was statistically significant for the THI global score and for 2 of the 3 subscales (Emotional and Catastrophic). The interaction effect for the THI global score was *F*_2,132_=6.15; *P*=.003; *e*^2^=0.085. After 3 months of using ReSound the THI global score significantly decreased in the study group (*P*<.001), and at the 6-month follow-up it was significantly lower in comparison with the initial level (*P*<.001). However, there was no statistically significant difference (*P*=.23) between the 3- and 6-month follow-ups. In the control group, scores were similar at the initial measurement and at both follow-ups.

The same was true for the Emotional subscale, where the interaction effect was statistically significant (*F*_2,132_=9.79; *P*<.001; *e*^2^=0.139). Again, 3 months of using ReSound resulted in a significant decrease of tinnitus severity in the study group (*P*<.001), and at the 6-month follow-up tinnitus severity was significantly lower in comparison with the initial level (*P*<.001). There was no statistically significant difference (*P*=.13) between the 3- and 6-month follow-ups. Tinnitus severity remained stable in the control group across all analyzed measurements.

The interaction effect for the Catastrophic subscale was statistically significant (*F*_2,132_=11.97; *P*<.001; *e*^2^=0.185). Multiple comparisons showed that tinnitus severity significantly decreased in the study group at the 3-month follow-up (*P*<.001) and again significantly decreased after another 3 months (*P*=.01). The scores on the Catastrophic subscale remained stable in the control group.

For the Functional subscale the interaction effect was statistically nonsignificant (*F*_2,132_=2.89; *P*=.06).

**Figure 1:**
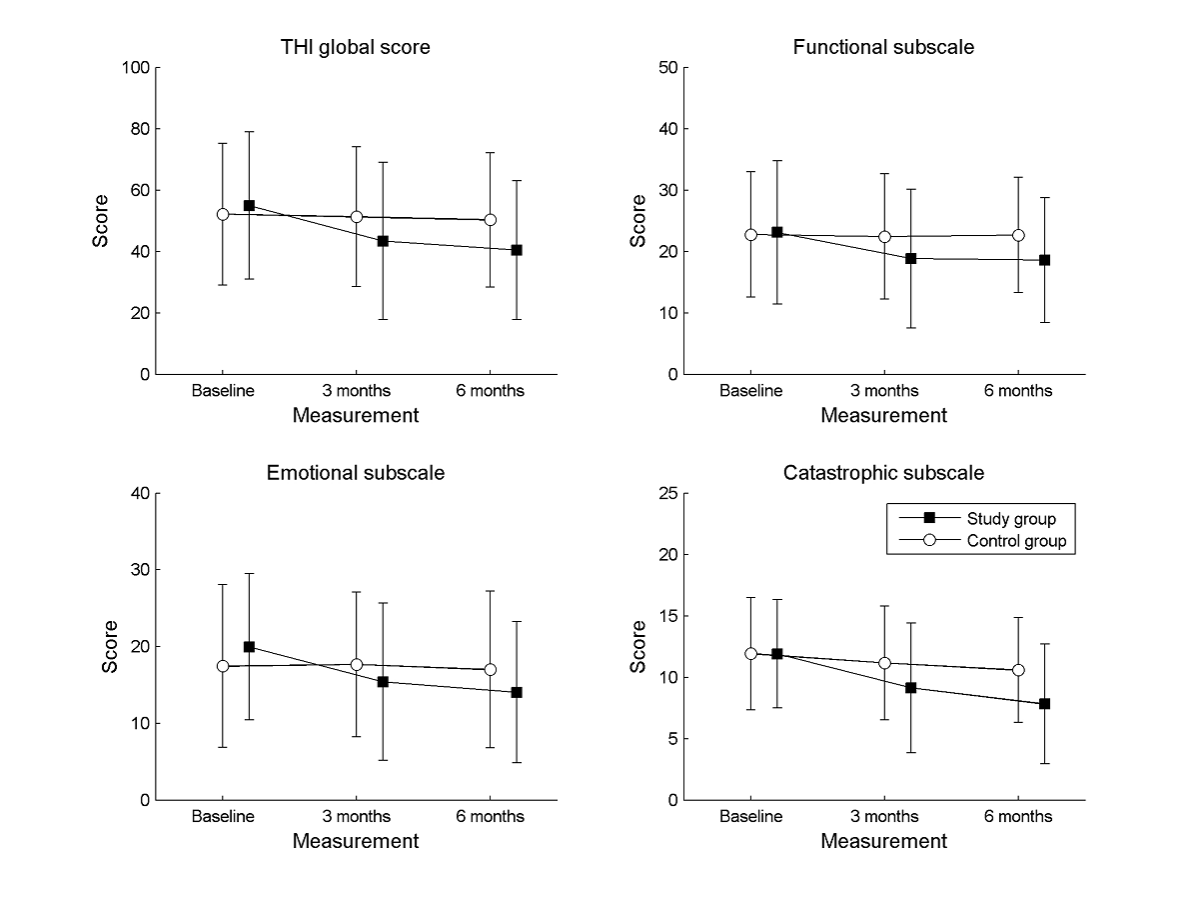
Mean scores obtained by the participants in the study and control groups on the THI. The squares and circles are mean scores, the error bars are SDs. THI: Tinnitus Handicap Inventory.

Mean scores for the THI in both groups and all measurements are shown in Figure 1.

**Table 2:** Study group results: descriptive statistics of tinnitus severity as measured with the THI subscale.

THI^a^ subscale and Measurement		Range	Mean (SD)	Q1^b^	Median	Q3^c^
**Functional**						
	Baseline	2-44	23.09 (11.67)	14.5	22	34
	3 months’ follow-up	0-44	18.84 (11.32)	10	17	26
	6 months’ follow-up	4-44	18.59 (10.15)	10	19	24
**Emotional**						
	Baseline	4-36	19.95 (9.53)	12	20	28
	3 months’ follow-up	0-36	15.41 (10.26)	8	16	21.5
	6 months’ follow-up	0-36	14.05 (9.20)	6.5	14	18
**Catastrophic**						
	Baseline	2-20	11.91 (4.40)	8	11	16
	3 months’ follow-up	0-20	9.14 (5.29)	6	10	14
	6 months’ follow-up	0-18	7.82 (4.88)	4	8	11.5
**THI global**						
	Baseline	14-100	54.95 (23.98)	36.5	55	76.5
	3 months’ follow-up	0-96	43.39 (25.60)	20.5	41	57.5
	6 months’ follow-up	6-92	40.45 (22.57)	18.5	42	54

^a^THI: Tinnitus Handicap Inventory.

^b^Q1: lower quartile.

^c^Q3: upper quartile.

**Table 3:** Control group results: descriptive statistics of tinnitus severity as measured with the THI subscale.

THI^a^ subscale and Measurement		Range	Mean (SD)	Q1^b^	Median	Q3^c^
**Functional**						
	Baseline	4-40	22.75 (10.18)	14.5	23	31.5
	3 months’ follow-up	0-40	22.42 (10.20)	18	23	28
	6 months’ follow-up	2-40	22.67 (9.37)	16	24	28
**Emotional**						
	Baseline	0-34	17.46 (10.60)	8	18.5	26
	3 months’ follow-up	2-32	17.67 (9.41)	8.5	19	26
	6 months’ follow-up	0-32	17.00 (10.22)	8.5	21	25.5
**Catastrophic**						
	Baseline	4-20	11.92 (4.59)	8	11	16
	3 months’ follow-up	0-18	11.17 (4.64)	6.5	12	15.5
	6 months’ follow-up	0-18	10.58 (4.27)	8	12	13.5
**THI global**						
	Baseline	12-92	52.13 (23.14)	31	52.5	69.5
	3 months’ follow-up	6-90	51.25 (22.74)	33	54	68
	6 months’ follow-up	6-94	50.25 (21.82)	36.5	50	66

^a^THI: Tinnitus Handicap Inventory.

^b^Q1: lower quartile.

^c^Q3: upper quartile.

### Clinically Important Change in Tinnitus Severity

At the 3-month follow-up a clinically significant improvement in tinnitus severity as shown with the THI (an improvement of ≥20 points) was found in 13/44 patients in the study group (30%) but in none of the controls. The difference between the groups was statistically significant (*χ*_1_^2^=8.77; *P*=.003). At the 6-month follow-up, a clinically significant improvement in the THI was found in 17/44 patients in the study group (39%) and again in none of the controls; the difference was statistically significant (*χ*_1_^2^=12.36; *P*<.001).

### Tinnitus Loudness and Annoyance as Measured With VAS

Descriptive statistics for the tinnitus loudness and tinnitus annoyance scores for initial and follow-up measurements in both the study and control groups are shown in Tables 4 and 5, respectively.

**Figure 2:**
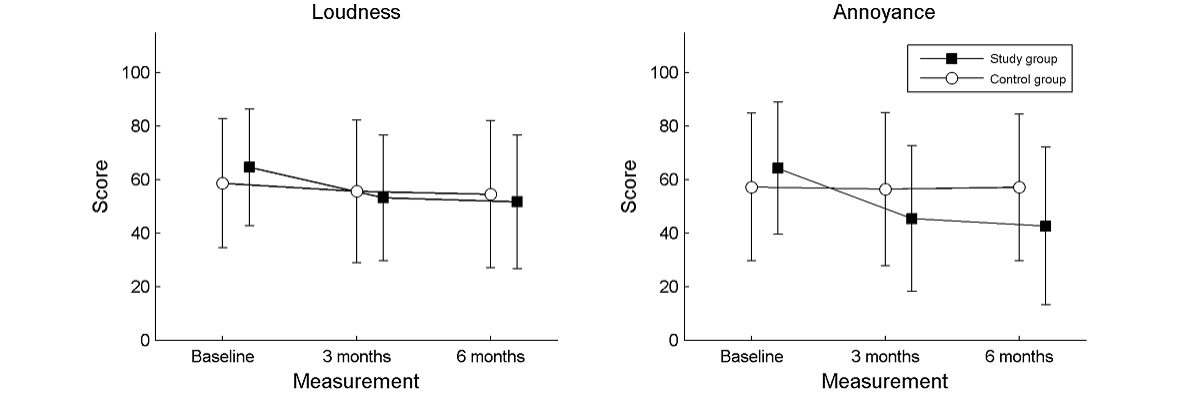
Mean scores obtained by the participants in the study and control groups on tinnitus loudness and tinnitus annoyance as measured by VAS. The squares and circles are mean scores, the error bars are SDs. VAS: visual analog scale.

For tinnitus loudness, the interaction effect (group × time) was statistically significant (*F*_2,126_=3.19; *P*=.04; *e^2^*=0.048) and multiple comparisons showed that tinnitus loudness at the 3-month follow-up had decreased significantly in comparison with baseline (*P*<.001). It was also significantly lower at the 6-month follow-up in comparison with baseline (*P*<.001), but there was no statistically significant difference (*P*>.99) between the 3- and 6-month follow-ups. Tinnitus loudness did not change significantly (*P*>.99) in the control group over 6 months. A similar but stronger effect was observed for tinnitus annoyance (*F*_2,126_=11.28; *P*<.001; *e*^2^=0.152). Mean scores for tinnitus loudness and tinnitus annoyance in both groups and all measurements are shown in Figure 2.

**Table 4:** Study group results: descriptive statistics of the results of tinnitus loudness and tinnitus annoyance as measured with VAS.

VAS^a^ and Measurement		Range	Mean (SD)	Q1^b^	Median	Q3^c^
**Loudness**						
	Baseline	20-100	64.55 (21.81)	50	66.5	80.8
	3 months’ follow-up	11-95	53.11 (23.44)	35.5	50.5	74.3
	6 months’ follow-up	0-95	51.66 (24.98)	32.8	51.5	75.5
**Annoyance**						
	Baseline	10-100	64.20 (24.72)	50.5	66.5	81
	3 months’ follow-up	0-97	45.43 (27.13)	23.3	45	67.3
	6 months’ follow-up	0-95	42.61 (29.45)	17.8	39.5	66.5

^a^VAS: visual analog scale.

^b^Q1: lower quartile.

^c^Q3: upper quartile.

**Table 5:** Control group results: descriptive statistics of the results of tinnitus loudness and tinnitus annoyance as measured with VAS.

VAS^a^ and Measurement		Range		Mean (SD)	Q1^b^	Median	Q3^c^
**Loudness**							
	Baseline	14	95	58.62 (24.13)	36	60	80
	3 months’ follow-up	6	98	55.57 (26.70)	37	51	80
	6 months’ follow-up	5	92	54.50 (27.47)	31.3	58.5	78
**Annoyance**							
	Baseline	3	97	57.19 (27.54)	38	60	80
	3 months’ follow-up	3	96	56.39 (28.58)	35	56	82
	6 months’ follow-up	3	91	57.04 (27.40)	35.5	63	80.8

^a^VAS: visual analog scale.

^b^Q1: lower quartile.

^c^Q3: upper quartile.

### Data Concerning the Use of ReSound by the Participants

**Figure 3:**
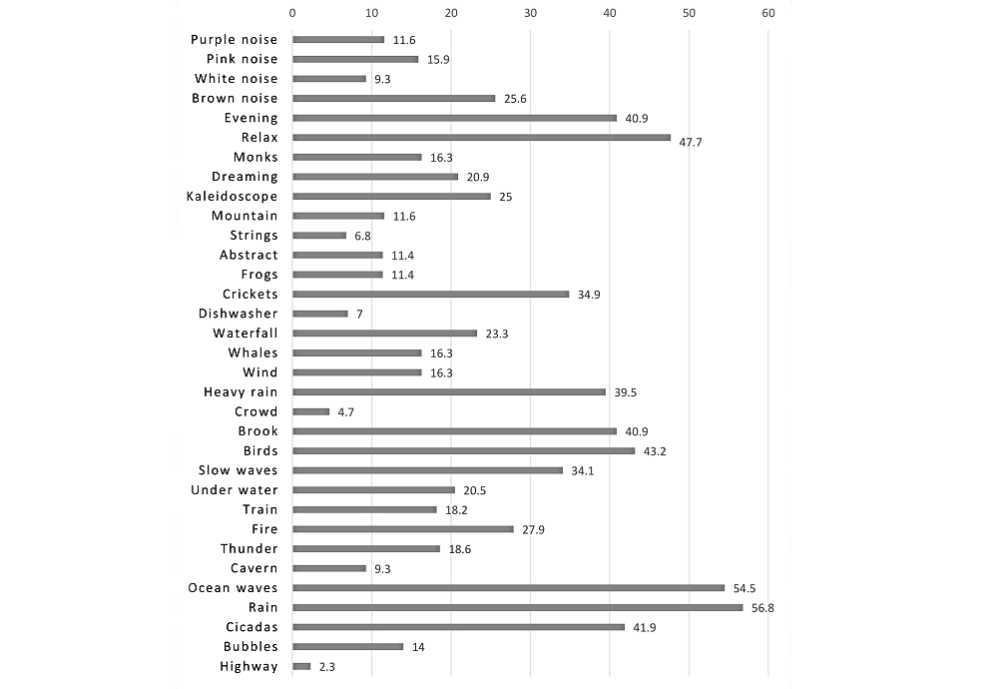
Sounds listened to by the participants.

A majority of study group participants (39/44, 89%) said they listened to environmental sounds, 43% (19/44) chose music, and 18% (8/44) therapeutic sounds. The most popular environmental sounds were rain and ocean waves, which were chosen by more than half of the participants. Detailed data on the sounds listened to are shown in Figure 3.

Of the 44 participants, 24 (55%) reported they used the app every day, 18 participants (41%) used it a few times a week, and 2 participants (5%) only once a week.

As many as 12 participants (27%) reported they used the app less than 30 minutes a day; 28 (64%) 1-2 hours a day, 3 (7%) 3-8 hours a day, and 1 (2%) used it for more than 8 hours a day.

As mentioned, the ReSound app also provides breathing and relaxation exercises and meditation support. Eight patients declared they had used other functions included in the app. Five of them used breathing exercises, 5 used the “Information” section, and 1 used the “Meditation” section (some people used >1 function). Therefore, in general, using additional functions was not very frequent. At the same time, participants knew that the main purpose of using the app was to listen to sounds, and they answered the surveys and questionnaires from this angle.

Participants rated the overall effectiveness of the app. Most of them (n=23, 52%) assessed it as moderate, 15 (34%) as high, 1 (2%) as very high, and 4 (9%) as low; 1 person did not answer the question.

## Discussion

### Principal Findings

This study showed that using a mobile app that generates sounds reduces tinnitus severity. An improvement was found especially in the emotional domain. Furthermore, the patients accepted the app and its overall rating was positive.

Based on the general results of the THI questionnaire, tinnitus severity in the study group decreased significantly, whereas it remained unchanged in the control group. The overall mean tinnitus severity in the study group decreased from 54.95 to 40.45 points, whereas in the control group it remained stable (mean values: 52.13 and 50.25 points, respectively). Additionally, we found a clinically significant improvement in 17/44 patients (39%) who used the mobile app for 6 months. A similar observation was made by Henry et al [24] who tested the app in a group of 25 people with tinnitus for 8 weeks and found that almost one-third of them (8/25) reported a significant improvement. Similarly, in our pilot study [26], a clinically significant change (as calculated based on the THI questionnaire scores) was reported in 54% of our participants (n=28).

The observed reduction in tinnitus severity may be due to many mechanisms. Some were offered by Munir and Pryce [37,38], who observed that listening to pleasant sounds allowed patients to escape from a problem by providing a diversion from the intrusiveness of tinnitus. The sounds also stimulated pleasant memories and allowed a break away from reality. Another possible explanation is that the sound enrichment devices are a way of having control over tinnitus. The smartphone and its app can be regarded as a tool that enables control over an invisible condition. A study by Dauman and Dauman [39] suggested that deciding what sounds to listen to created a sense of control over tinnitus and reduced the mental effort required to distract oneself from tinnitus. They also suggested that controlling tinnitus using an external tool had a positive effect on self-empowerment and improved psychological functioning of the patient [39].

Our results show that the highest improvement in tinnitus severity was found on the Emotional subscale. This subscale identifies emotions and states that accompany tinnitus such as anger, frustration, worry, irritability, and perhaps depressive symptoms [40]. A statistically significant improvement was also found on the Catastrophic reaction subscale, which assesses reactions a patient typically encounters when tinnitus arises: feelings of desperation, enslavement, seeing it as a terrible disease, and the sense of lack of control [40]. A reduction in the emotional burden of tinnitus also may be reflected in the changes measured by the second tool, the VAS. We saw a significant improvement (VAS scores) in both loudness and annoyance in the study group, but the effect was stronger for annoyance than for loudness. By comparison, in the control group, both measures were stable over 6 months. It should be noted that in our study a change in tinnitus severity was not found for the Functional subscale. This means that the participants always felt a limitation due to their tinnitus in the areas of cognitive, social, and physical functioning. The advantage of the app was that it led to lower negative emotions and thus reduced overall tinnitus severity.

When the patients’ preferences for the type of sound to listen to were assessed, almost 90% (39/44) listened to environmental sounds. The most popular sounds were rain and ocean waves. These results are consistent with those of Henry et al [24] who found that participants preferred the sounds of ocean waves and rain. In the study by Tyler et al [25], the sounds most frequently listened to and enjoyed were rain and waves on rocks, and for Perreau et al [28] heavy rain, pink noise, and waterfalls.

The usage survey showed that most of the participants used the app every day and usually for 1-2 hours a day. However, the data were based only on self-reports and we were unable to confirm them. In its present form, the app does not allow the duration of use to be recorded. This is a limitation that might be overcome in future research by providing a data logging function that could track and store user activity. Another limitation of the study is the appreciable number of participants who dropped out. The data were collected by postal mail and coincided with the outbreak of COVID-19 [41]. The introduction of a national lockdown and other restrictions reduced the ease with which patients could send in data. In the future, it would be better to collect data electronically, for example, using an online platform that ensures a full set of results.

It should be also mentioned that when treatment is applied, patients could be influenced by placebo effects. Therefore, it is worth considering the use of a technical placebo for the control group in future research. Unfortunately, in the present setting it was not possible. The app used is a commercial product, and we did not have the possibility to introduce any changes to the generated sounds.

Taken together, however, using a mobile app for tinnitus sound therapy does seem to be a promising solution. In future work, research in a more controlled environment is needed. App developers might consider implementing a system that allows data to be logged, as well as tracking the time and mode of app usage. This would enable a better understanding of how patients interact with the app and document their patterns of usage.

### Conclusions

Use of an app that generates background sounds appears to be an effective way of reducing tinnitus severity. Although participants still experienced limitations caused by tinnitus in the areas of cognitive, social, and physical function, the advantage of the app was that it led to lower negative emotions and thus reduced overall tinnitus severity. It is worth considering whether mobile apps might be usefully employed to manage tinnitus in a professional setting.

## Abbreviations

TFI: Tinnitus Functional Index
THI: Tinnitus Handicap Inventory
VAS: visual analog scale
